# Neonatal stress-induced affective changes in adolescent Wistar rats: early signs of schizophrenia-like behavior

**DOI:** 10.3389/fnbeh.2014.00319

**Published:** 2014-09-10

**Authors:** Carlos Eduardo Neves Girardi, Natália Cristina Zanta, Deborah Suchecki

**Affiliations:** Department of Psychobiology – Escola Paulista de Medicina, Universidade Federal de São PauloSão Paulo, SP, Brazil

**Keywords:** early life stress, animal model, schizophrenia, corticosterone, anxiety-like behavior, social behavior, anhedonia

## Abstract

Psychiatric disorders are multifactorial diseases with etiology that may involve genetic factors, early life environment and stressful life events. The neurodevelopmental hypothesis of schizophrenia is based on a wealth of data on increased vulnerability in individuals exposed to insults during the perinatal period. Maternal deprivation (MD) disinhibits the adrenocortical response to stress in neonatal rats and has been used as an animal model of schizophrenia. To test if long-term affective consequences of early life stress were influenced by maternal presence, we submitted 10-day old rats, either deprived (for 22 h) or not from their dams, to a stress challenge (i.p. saline injection). Corticosterone plasma levels were measured 2 h after the challenge, whereas another subgroup was assessed for behavior in the open field, elevated plus maze (EPM), social investigation and the negative contrast sucrose consumption test in adolescence (postnatal day 45). Maternally deprived rats exhibited increased plasma corticosterone (CORT) levels which were higher in maternally deprived and stress challenged pups. Social investigation was impaired in maternally deprived rats only, while saline injection, independently of MD, was associated with increased anxiety-like behavior in the EPM and an impaired intake decrement in the negative sucrose contrast. In the open field, center exploration was reduced in all maternally-deprived adolescents and in control rats challenged with saline injection. The most striking finding was that exposure to a stressful stimulus *per se*, regardless of MD, was linked to differential emotional consequences. We therefore propose that besides being a well-known and validated model of schizophrenia in adult rats, the MD paradigm could be extended to model early signs of psychiatric dysfunction, and would particularly be a useful tool to detect early signs that resemble schizophrenia.

## Introduction

Mental illnesses are among the most frequent causes of functional debilitation. Schizophrenia is a markedly devastating psychiatric disorder, affecting approximately 1% of the global population (Freedman, 2003). It is challenging to precisely outline the causes of schizophrenia, because a multitude of genetic and environmental factors are thought to interact in the course of the disease, resulting in the onset, maintenance and evolution of schizophrenic symptoms (for a review on the multifactorial aspect of mental disorders, see Akdeniz et al., 2014; Uher, 2014).

One of the most explored aspects on the environment-gene interaction prompting schizophrenic phenotype is the impact of harsh early life conditions, leading to the neurodevelopmental hypothesis of schizophrenia (Fatemi and Folsom, 2009; Piper et al., 2012), which postulates that neurochemical disturbances induced by adverse early life events could permanently affect brain function, producing abnormalities that would ultimately underlie the emergence of the disorder. The early stages of life are characterized by an intense neural development, in which there is a dynamic process of synaptic shaping and pruning, making this period highly vulnerable to damaging disturbances (Martínez-Téllez et al., 2009; Stolp et al., 2012).

The impact of childhood experiences on the development of emotional behavior and their influence on psychopathology has received vigorous scientific support in the past decades (Heim et al., 1997, 2000; Bernet and Stein, 1999; Heim and Nemeroff, 2002; McEwen, 2003). However, studies that aim at correlating early life events to schizophrenia or to other psychopathologies are rather speculative and based on retrospective subjective reports. Manipulations of the early environment for subsequent assessment of emotional state in humans are operationally and ethically unfeasible. Therefore, animal models are imperative to comprehend how early life adverse events affect behavioral and neurobiological aspects that reflect the basic underpinnings of mental disorders such as schizophrenia.

Developmental stress models used to investigate long-lasting consequences on schizophrenia-related emotional and cognitive behavior range from prenatal maternal immune (Meyer et al., 2006) and psychological challenges (Kinnunen et al., 2003; Lee et al., 2007), to maternal vitamin D deficiency (Kesby et al., 2006), maternal deprivation (MD; Ellenbroek et al., 1998; Garner et al., 2007) and socially unstable conditions (Möller et al., 2011). An acute 24-h MD on post-natal day (PND) 9 exerts profound effects on a myriad of affective and cognitive features in the offspring that have been linked to major processes underlying clinical manifestations of schizophrenia (Ellenbroek et al., 1998; Ellenbroek and Cools, 2002b; Ellenbroek et al., 2005; Takase et al., 2012). The rationale for the putative MD effects is the fact that during the first 2 weeks of life, the infant hypothalamus-pituitary-adrenal (HPA) axis shows a blunted basal activity as well as a marked hyporesponsiveness to external noxious stimuli (Sapolsky and Meaney, 1986; Rosenfeld et al., 1992; Schmidt et al., 2003). This period is named stress hyporesponsive period (SHRP) and core elements of the maternal care were identified as pivotal for SHRP maintenance (Cirulli et al., 1992; Suchecki et al., 1993). Prolonged maternal absence disinhibits basal and reactive adrenocortical activity to mild stressors, producing high circulating plasma level of corticosterone (CORT) during a stage when it is naturally upheld low (Levine et al., 1991; Suchecki et al., 1995; Faturi et al., 2010). Despite the strong body of evidence showing that high circulating levels of CORT can affect brain structures that orchestrate cognitive and emotional behavior (Woolley et al., 1990; Mitra and Sapolsky, 2008), it is not yet completely understood whether CORT action on neural development is the mechanism involved in the long-lasting MD effects that resemble symptoms of schizophrenia.

The DSM-V lists three main clusters of signs that are required for schizophrenia diagnosis, comprising the so-called positive, negative and cognitive symptoms (American Psychiatric Association, A.P.A.D.S.M.T.F., 2013). Although it is puzzling to mimic hallucinatory and delusional behaviors in animal studies, positive symptoms of schizophrenia are still easily modeled in animals because pharmacological validation tools can be used to investigate rodent analogs of psychosis (such as prepulse inhibition (PPI) impairments and hyperlocomotion). Because overactive dopamine is a key-candidate mechanism to underpin positive symptoms (Howes and Kapur, 2009; Lodge and Grace, 2011), animal models that aim to mimic these symptoms evaluate behaviors that are mainly affected by a magnification of the dopaminergic transmission, such as hyperlocomotor activity in novel environments (Powell et al., 2009; Le Pen et al., 2011) and hypersensitivity to dopaminergic drugs (Ellenbroek and Cools, 2002a). Also extensively employed are animal models that explore the negative symptoms, which include affective flattening, anhedonia, avolition and asociality (Ellenbroek and Cools, 2000; Moser, 2014).

By all means, most of the developmental interventions aim at behavioral assessments that have been considered to have specific translational relevance to schizophrenia. Evaluation of a more comprehensive range of emotional behaviors would help clarify whether the abovementioned effects of MD are restricted to schizophrenic-like features that emerge in adulthood or if there is an overall effect on a broader spectrum of emotionality that manifests likewise earlier in life. Furthermore, these aspects are typically tested in early adulthood, when the schizophrenic-like phenotype is assumed to be fully established. However, the initial signs may include poor sociability and anxiety symptoms followed by the onset of typical psychotic outbursts (Agius et al., 2010). Hence, aside from testing typical schizophrenia-like behavior restricted to adulthood, current investigation approaches should expand the assortment of behaviors and anticipate the age of interest, ensuring that early alterations such as augmented anxiety and mild social impairment in adolescence would be included in the scope of developmental animal models of schizophrenia.

One additional step for a full characterization of MD as an unambiguous developmental animal model for schizophrenia would be to match the effects of MD to the effects of different early life stressors. If one could dissociate the effects of maternal care disruption and that of different stressors, this would pave the way to the understanding on how specific the association is between MD and schizophrenia-like phenotype in animal models. Alternatively, if disruption of maternal care prompts the HPA axis to a reactive state, adding an extra stress challenge to the maternally-deprived infant rat, with an external noxious stimulus, would trigger a robust neuroendocrine response, which, in turn, could be responsible for more intense and impacting developmental and behavioral effects.

Taken together, the aforementioned evidence indicates that experimental approaches of concurrent early life events and earlier characterization of behavioral effects are necessary to better illustrate the time course and the full range of symptomatology witnessed in the clinical scenario. Therefore, we employed the MD paradigm and a mild stress challenge or a combination of both to characterize, in adolescence, a wide range of behavioral effects that might unravel early signs of psychiatric dysfunction in this developmental stress animal model.

## Materials and methods

### Subjects and experimental design

Fifteen pairs of Wistar rats were bred, generating a total of 96 male and 24 female Wistar rat pups, of which, only males were used in this study. Male-female pairs were obtained from the Center for the Development of Animal Models for Biology and Medicine (CEDEME), Universidade Federal de Sao Paulo. All procedures were approved by, and conducted in accordance with the Research Ethics Committee of the Universidade Federal de Sao Paulo, approval protocol #0366/12.

Litters were obtained from mating pairs maintained together in Plexiglas cages under controlled temperature (22 ± 2°C) and a light-dark cycle of 12 h, with lights on at 7 a.m. 10 days after the onset of breeding, couples were separated and females were individually housed until the end of experiments. 15 days after the onset of breeding, sawdust and paper towels were provided for nest building. Inspection for births started on day 18 and continued twice daily, at 9:00 a.m. and at 5:00 p.m. The day of birth was considered postnatal day 0 (PND 0) and litters were culled to approximately 6 males and 2 females, whenever possible, on PND 1. When the number of newborns in a litter did not reach the minimal of 6 males or 2 females, additional males or females (when available) were maintained at culling or surplus pups were adopted from litters born in the same day, in order to preserve the standard number of 8 pups per litter. Litters with less than 8 pups were not used in the experiment (*n* = 3 litters). Births occurred in a range of 4 days (22–25 days after mating onset). The 12 remaining litters were assigned to Maternal Deprivation (MD = 6 litters) or Control non-deprived (CON = 6 litters) groups. For MD, the dam was removed from the home-cage and housed in a separate room for 24 h, from PND 9 to 10. CON litters were left undisturbed, with their mothers, during this period. Twenty-two hours after the onset of MD, or at the corresponding time for CON litters, each group was subdivided in two: a subgroup was submitted to a stress challenge, consisting of an intraperitoneal injection of 0.3 mL of a 0.9% saline solution (STR) and another subgroup was kept unstressed, with no saline injection (UNS). UNS pups were briefly handled in the same room in which injections occurred. In order to counterbalance birth date among groups, each new delivery was assigned *a priori* to one of the groups, in the following order: CON UNS (3 litters), CON STR (3 litters), MD UNS (3 litters) and MD STR (3 litters). Two hours after the saline injections, 1–2 pups/litter were decapitated and trunk blood was collected for determination of plasma CORT levels. Additional litters were included in this phase in order to increase the number of blood samples for acute blood analysis. The remaining pups returned to their mothers and were left undisturbed until weaning and cages were then cleaned every 4 days, so that half of the bedding material was removed and replaced with fresh one and nesting material was supplemented as necessary. On PND 21 (weaning), male offspring was individually identified and transferred to another animal room, under the same environmental conditions as described above, housed in regular Plexiglas cages in groups of 2–3 siblings per cage. Behavioral testing started on PND 45. One day before the onset of behavioral evaluation, each rat was individually housed with water and food ad libitum.

### Determination of plasma CORT levels

Trunk blood was collected in cooled EDTA-containing vials and centrifuged at 2300 rpm, 4°C for 15 min. Plasma was stored at −20°C for determination of CORT levels by radioimmunoassay, using a commercial kit for rats and mice (MP Biomedicals, Orangeburg, NY, USA) with a modification of the original method, using a reduced sample volume of 5 μl (Thrivikraman et al., 1997). The sensitivity of the assay was 3.125 ng/ml and intra and inter-assay variations were, respectively, 7.1% and 10.3%.

## Behavioral tests

### Sucrose negative contrast test (SNCT)

To test the intake response to a sudden decrease in concentration of a palatable sucrose solution, two identical bottles were provided for each single-housed rat, during 3 days. One bottle contained 200 mL of sucrose solution and the other an equal volume of water. On each day, bottle positions were interchanged to avoid an effect of side preference. Each bottle was weighed every 24 h (at 11:30 a.m.) to estimate liquid intake. On testing days 1 and 2, sucrose solution concentration was 15% and on day 3, it was 2.1% (Matthews et al., 1996). The sucrose preference index was calculated as the percentage of sucrose intake in relation to the total intake of liquid, according to the equation: Sucrose preference index = (sucrose intake/(water intake + sucrose intake)) × 100.

### Open field

On the afternoon after the end of the SNCT (from 12:00 p.m.–16:00 p.m.), each rat was individually placed in the center of a circular arena (80 cm diameter) surrounded by 50-cm high walls, and left undisturbed for 10 min. The luminosity during the test was limited to the lights placed on the top of the open field (25 lux). The arena was thoroughly cleaned with 70% ethanol between animal sessions to eliminate olfactory cues. The tests were recorded using a digital video camera and an off-line analysis was performed using the software Ethovision XT (Noldus, The Netherlands). The parameters used were distance and time traveled in each virtually defined compartment (center and periphery) as well as total ambulated distance by each rat. Center was defined as a 52-cm diameter (14 cm from the wall) concentric inner circle.

### Elevated Plus Maze (EPM)

In the next morning after open field test (from 9:00 a.m.–12:00 p.m.), rats were tested on the Elevated Plus Maze (EPM). The maze is elevated 60 cm above the floor and consists of four arms: two of them are enclosed by 40 cm high walls, and the other two are open arms (with no enclosing walls). Each arm is 50 cm long and 10 cm wide. Procedures were based on those described elsewhere (Pellow et al., 1985). Briefly, each animal was individually placed in the central segment of the maze facing one of the open arms, interchangeably on consecutive animals. Each test session lasted 5 min. The apparatus was thoroughly cleaned with 70% ethanol between sessions. The tests were carried out in a dim environment (11 lux). We manually scored the number of entries and the time spent in open and closed arms. Data are expressed as percentage of time and percentage of entries in the open arms.

### Social investigation

In the afternoon of the same day as the EPM test (from 12:00 –16:00 p.m.), rats were submitted to the social investigation test, which was conducted in the same arena and same environmental conditions as the open field test so that the animals were familiarized to the environment. Inside the arena two identical metal grid cylindrical cages (20 cm in diameter and 25 cm in height) were placed on opposite sides of the arena. One cage was empty and the other contained a naïve rat. Each experimental animal was individually placed in the center of the arena and left undisturbed to explore the environment for 10 min. Tests were recorded using a digital video camera and an off-line analysis was manually performed. Cage exploration was computed when the rat approached and pointed its nose towards one of the cages and as close as possible to the cage (approximately 1 cm or as closer as it was possible to judge from the video image). We quantified the time of exploration of the empty cage and the cage containing the naïve rat.

### Statistical analysis

Each parameter was analyzed by two-way ANOVA, with Maternal Deprivation (CON vs. MD) and Saline Injection (Unstressed [UNS] vs. Stressed [STR]) as main factors. The SNCT was analyzed by repeated measures ANOVA, in which the within subject factor was Day (day 1 vs. day 2 vs. day 3) and MD and Saline Injection as between factors. The level for statistical difference was set at *p* < 0.05. When a significant main effect or an interaction was detected, the Newman-Keuls *post hoc* test was applied for between-group comparisons.

## Results

### CORT plasma levels

**Figure 1:**
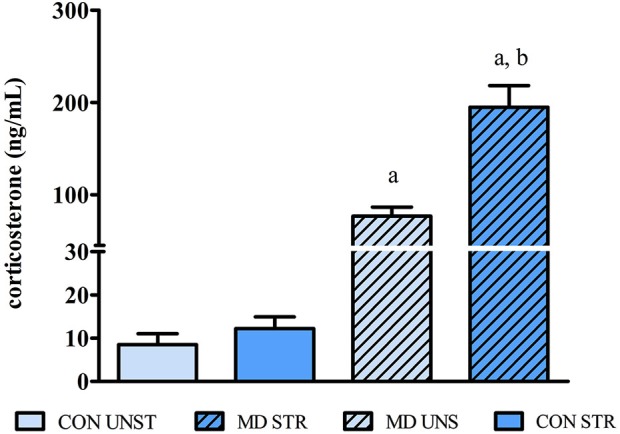
**CORT plasma levels on PND10 (24 h after maternal deprivation onset/2 h after saline injection) expressed as mean ± S.E.M.** CON = group not maternally deprived; MD maternal deprivation on postnatal day 9. UNS = no stress challenge; STR = saline injection stress challenge. a = significantly different from respective CON; b = significantly different from respective UNS (*n* = 11–16/group).

There was a significant effect of MD factor (*F*_(1,50)_ = 64.767, *p* < 0.001), and Saline Injection (*F*_(1,50)_ = 15.215, *p* < 0.0003) as well as an interaction between these factors (*F*_(1,50)_ = 13.418, *p* = 0.0006). *Post-hoc* analysis revealed that MD increased plasma CORT levels (CON UNS vs. MD UNS, *p* = 0.008) and this effect was further increased by the saline injection (MD UNS vs. MD STR, *p* = 0.0001) (Figure 1).

### Sucrose negative contrast test

**Figure 2:**
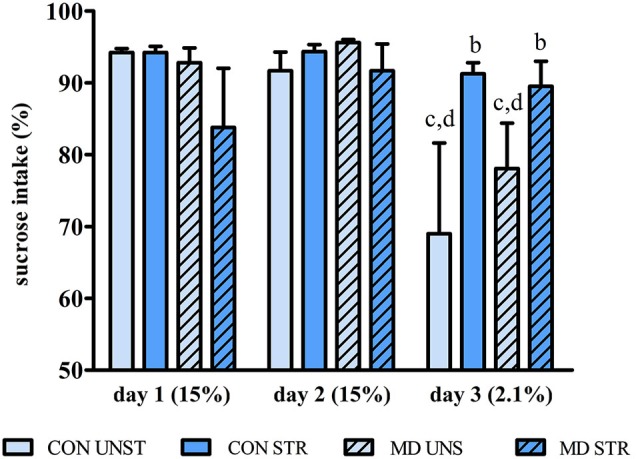
**Mean ± S.E.M. percentage of sucrose intake during the course of 3-day testing relative to total liquid intake in each day**. CON = group not maternally deprived; MD maternal deprivation on postnatal day 9. UNS = no stress challenge; SAL = saline injection stress challenge. b = significantly different from respective UNS; c = significantly different from respective intake on day 1; d = significantly different from respective intake on day 2 (*n* = 8–10/group).

UNS, but not STR animals, drank less 2.1% than 15% sucrose solution (day 3 < [day 1 = day 2 (*p*’s < 0.005)]); effect of day *F*_(2,52)_ = 7.395, *p* = 0.001). When the low concentration of sucrose was offered, STR rats drank more than UNS rats, regardless of MD (interaction between Day and Saline Injection (*F*_(2,52)_ = 7.107, *p* < 0.002; UNS vs. STR on Day 3, *p* < 0.03) (Figure 2).

### Open field

**Figure 3:**
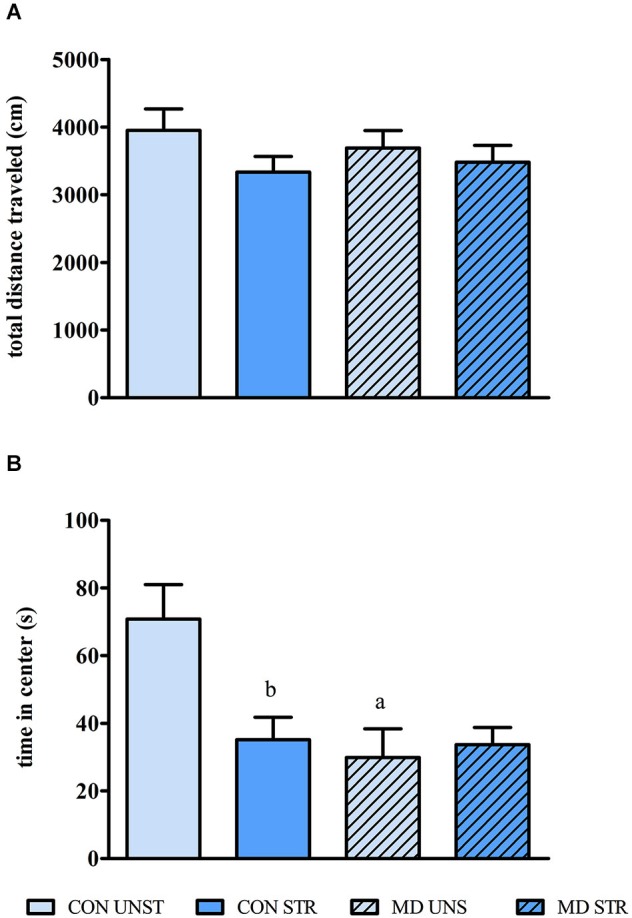
**Mean ± S.E.M. total distance traveled (A) and time spent in the central region of the open field (B)**. CON = group not maternally deprived; MD maternal deprivation on postnatal day 9. UNS = no stress challenge; SAL = saline injection stress challenge. a = significantly different from respective CON; b = significantly different from respective UNS (*n* = 8–10/group).

Ambulation, measured as total distance traveled in the open field, was not affected neither by MD (*F*_(1,30)_ = 0.053, *p* = 0.818) nor by saline injection *F*_(1,30)_ = 2.253, *p* = 0.143) (Figure 3A). Conversely, there was a clear effect of the interaction between MD and Saline Injection for both distance traveled and time spent in the center portion of the open field (Distance traveled: *F*_(1,30)_ = 5.860, *p* = 0.021 and time spent in the center *F*_(1,30)_ = 5.848, *p* = 0.021). CON STR and MD UNS groups ambulated significantly less (*p*’s = 0.012) and spent less time (Figure 3B) in the center of the open field than CON UNS rats (*p* < 0.015 and *p* < 0.009, respectively).

### Elevated plus maze

**Figure 4:**
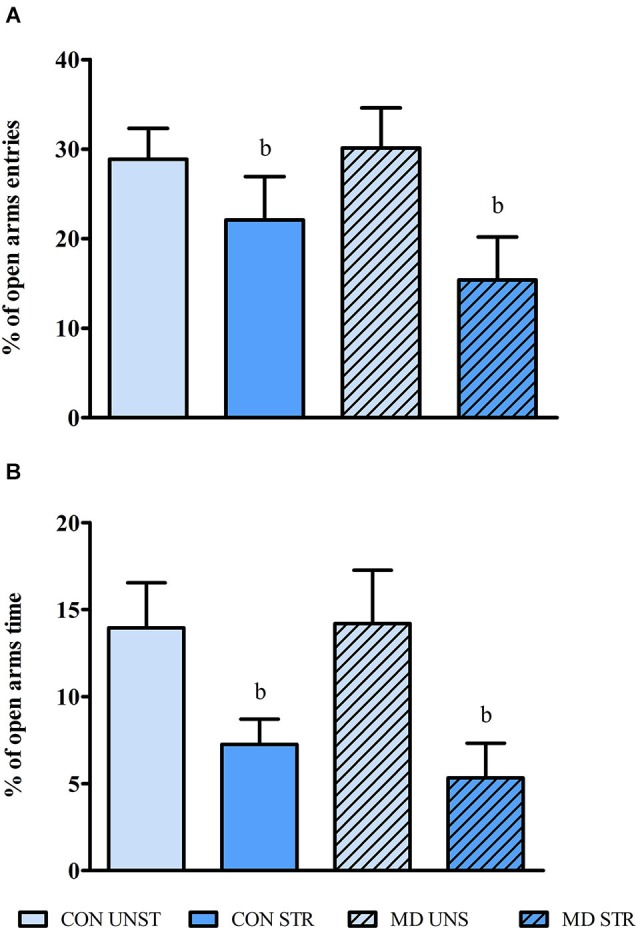
**Mean ± S.E.M. percentage of open arms entries (A) and of open arms time (B) in the EPM test**. CON = group not maternally deprived; MD maternal deprivation on postnatal day 9. UNS = no stress challenge; SAL = saline injection stress challenge. b = significantly different from respective NSAL (*n* = 8–10/group).

Rats that received a saline injection on PND 10 entered less and spent less time in the open arms than their UNS counterparts (main effect of Saline Injection for percentage of entries (*F*_(1,30)_ = 5.928, *p* < 0.020 and for percentage of time in the open arms *F*_(1,30)_ = 9.761, *p* < 0.004, respectively). No significant effect was observed on the percentage of closed arms exploration time or number of closed arm entries, indicating that no effect on locomotor activity on the EPM was observed (Figure 4).

### Social investigation

**Figure 5:**
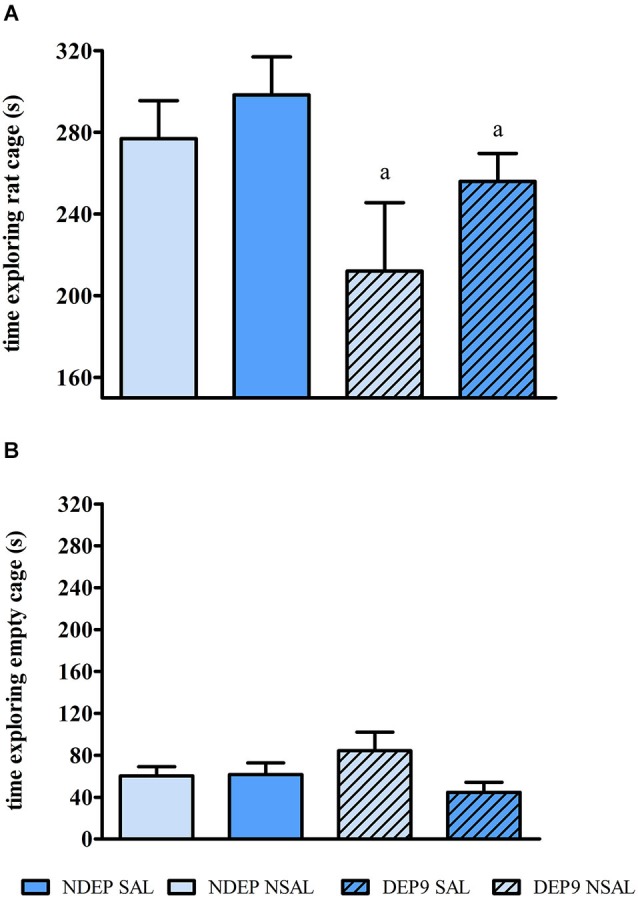
**Mean ± S.E.M. time exploring the rat-containing cage (A) and the empty cage (B) on the social investigation test**. CON = group not maternally deprived; MD maternal deprivation on postnatal day 9. UNS = no stress challenge; SAL = saline injection stress challenge. a = significantly different from respective CON (*n* = 8–10/group).

Rats submitted to MD spent less time investigating the rat-containing cage than CON rats (main effect of MD (*F*_(1,30)_ = 4.812, *p* < 0.04)) (Figure 5A), whereas no significant difference was found on the exploration of the empty cage (Figure 5B). No significant saline injection (*F*_(1,30)_ = 1.795, *p* = 0.190) nor an interaction between these two factors (*F*_(1,30)_ = 0.211, *p* = 0.648) was observed.

## Discussion

The present results confirmed previous findings that 24 h MD disinhibits basal and reactive adrenocortical activity to mild stressors, such as a saline injection (Levine et al., 1991; Suchecki et al., 1995; Faturi et al., 2010). The middle-term effects of MD included reduced ambulation in the center part of the open field and less social interaction than non-deprived rats. On the other hand, the saline injection on PND10 resulted in increased anxiety-like behavior and impairment in the sucrose negative contrast test (SNCT). Interestingly, there was no evidence of interaction between these two early events, inasmuch as MD STR neonates presented a four-fold increase in CORT levels after the saline injection compared to MD UNS rats, but behaviorally these groups exhibited similar alterations.

The open field test is useful to assess the locomotor activity and, in the case of animal models of schizophrenia, motor hyperactivity presents face (Powell et al., 2009) and construct validity (van den Buuse, 2010) to the positive symptoms, reflecting exaggerated release of dopamine in the mesolimbic pathway (Powell et al., 2009; Le Pen et al., 2011). This apparatus is also employed for the assessment of anxiety-like behavior, which is translated by reduced ambulation in the center portion of the arena (Miller et al., 2002), reduced vertical exploration, increased grooming and defecation (Denenberg, 1969; Masur et al., 1980). The results of the open field test indicated that MD was linked to an increased anxiety-like behavior, but surprisingly, a single saline injection in CON 10 day-old pups was also related to a similar behavioral alteration. These results are not in complete consonance with those observed in the EPM, a widely used apparatus to test anxiety-like behavior in rodents. In this test, the fewer the entries and the shorter the time spent in the open arms is interpreted as higher expression of anxiety-like behavior (Pellow et al., 1985). This test shows predictive validity for it is sensitive to anxiolytic drugs that act on GABA_A_ receptors, such as benzodiazepines, increasing the visits and time spent in open arms (Pellow and File, 1986). We observed that saline injection on PND10, regardless of maternal deprivation-induced sensitized CORT response, led to greater avoidance of the open arms, being thus, considered an anxiogenic stimulus. This outcome suggests that increased anxiety during adolescence is independent of the magnitude of the CORT response to stress in infancy, but that saline injection may activate other systems, as has been shown with restraint stress-induced CRH and AVP mRNA levels in the paraventricular nucleus of the hypothalamus (Dent et al., 2000a,b) and of CRH in the septum (Vazquez et al., 2006) during the SHRP. Therefore, even in the absence of a pituitary-adrenal response to stress, the central component of the HPA axis is active during the neonatal period and does not seem to require a previous disinhibition. Regarding the effect of MD on anxiety-like behavior, the present results replicate previous studies, which have shown that adolescent rats submitted to MD at PND9 do not display increased anxiety-like behavior in the EPM (Marco et al., 2013). However, the fact that PND11 maternally-deprived adolescents display greater exploration of the center part of the open field (Suchecki et al., 2000) and a similar exploration of the open arms in the EPM than their non-deprived counterparts (unpublished data), strongly suggests an age-dependent effect of MD.

Suppression of reward seeking by chronic stress is a well-known phenomenon (Anisman and Zacharko, 1982). This characteristic behavioral change has been used as an index of anhedonia (Muscat and Willner, 1992), a feature that reflects the lack of motivation to engage in pleasurable activities, which represents one of the core symptoms of depression (Willner et al., 1992). The negative contrast sucrose test has been used to test motivational behavior and is based on the fact that reductions in expected incentives lead to behavioral adjustments characterized as depression effects (reduced motivation), that reflects anticipatory frustrative emotionality (Amsel, 1958). Therefore, when animals perceive the shift in the salience from a highly (15% sucrose solution) to a less rewarding stimulus (2.1% sucrose solution), there is a reduction in consummatory behavior, which represents a hedonic feature (Verma et al., 2010). It has being shown that adult male and female rats submitted to maternal separation present smaller contrast, i.e., variation of intake from the 15% to 2.1% sucrose solution, than their respective control rats (Matthews et al., 1996). The diminished responsiveness to changing reward salience of STR groups, irrespective of MD, could be interpreted as an analog of the blunted hedonic responsivity seen in human depression (Matthews et al., 1996). However, evaluation of predictive validity is required prior to further speculation. Alternatively, the results may be interpreted as (1) an inability of saline injected adolescents to attribute differential salience values to different concentrations of the sucrose solution, thus higher or lower concentrations would represent the same rewarding value; or (2) these animals attributed higher palatable value to the stimulus, likely reflecting an anxiety trait, in which rats maintain the sucrose intake to alleviate this affective state. This supposition is corroborated by findings that increased intake of sucrose reduces corticotropin releasing factor mRNA in the paraventricular nucleus of the hypothalamus, suggesting that carbohydrates can counterbalance augmented activity of the stress response (Dallman et al., 2003). Reduction of the HPA axis activity could be a mediator of the anxiolytic-like effect of sucrose intake, since rats genetically selected for high levels of anxiety exhibit exacerbated HPA axis stress response (Wigger et al., 2004). Conversely, it has been shown that benzodiazepine anxiolytic drugs induce faster recovery from the suppressed intake after the downward shift in sucrose concentration (Becker, 1986; Flaherty, 1990). However in the present study we did not see the expected downward shift in sucrose intake in STR rats.

Schizophrenia is a psychiatric disorder that emerges in stages, with a typical course of symptoms, beginning with nonspecific clinical features, including depression, anxiety, social isolation and school/occupational failure. The early phase, which manifests in pre-adolescence until young adulthood is also marked by comorbid anxiety disorders, including panic and social anxiety, although the comorbidity has been overlooked for many years (Pallanti et al., 2013). According to the DSM-V, diminished emotional expression, anhedonia, asociality and avolition are prominent negative symptoms of schizophrenia and mild forms of such altered behaviors may manifest early in life (for review, see Azorin et al., 2014; Foussias et al., 2014). These behavioral alterations are relatively feasible to model in animals, particularly social interaction tests that have been widely employed (Moser, 2014). Among the several animal models of schizophrenia-like behavior, based on the neurodevelopmental hypothesis (Fatemi and Folsom, 2009), there seems to be a general consensus of reduced social interaction in adolescent animals, represented by increased latency to begin and shorter time engaging in social contact (Shi et al., 2003), reduced active interaction (Flagstad et al., 2004) and less time in contact (Blas-Valdivia et al., 2009), very much in consonance with the present results, in which a clear maternal deprivation-induced avoidance of social investigation of a naïve rat was observed. The fact that adult rats maternally deprived on PND9 exhibit a deficit of pre-pulse inhibition (Ellenbroek et al., 1998, 2005) prompted us to test these animals during adolescence, in order to explore if this paradigm could also serve to detect early signs that would predict a posterior emergence of schizophrenic-like traits. The present results pointed out to a broader spectrum of psychiatric dysfunction, ranging from a likely increase in anxiety-like traits, to reduced social interest, suggesting a lack of specificity as a schizophrenia model, while saline injection was linked to anxiety-like alterations, which can be viewed as a non-specific affective effect. On the other hand, it is still conceivable to propose the MD paradigm on PND9 as neurodevelopmental model of schizophrenia. MD was associated with impaired social interest, which is a valid approach for the negative symptoms of schizophrenia in rats (Moser, 2014) and this neurodevelopmental model is widely employed for investigating neurobehavioral alterations that resemble schizophrenic aspects in adult rats.

Taken together, our data support that the early emergence of MD-specific behavioral alterations can be regarded as a compelling tool to investigate brain changes and possible strategies of treatment or prevention in an earlier phase of schizophrenic-like features.

## Conflict of interest statement

The authors declare that the research was conducted in the absence of any commercial or financial relationships that could be construed as a potential conflict of interest.
